# A multicenter survey of first-line treatment patterns and gene aberration test status of patients with unresectable Stage IIIB/IV nonsquamous non-small cell lung cancer in China (CTONG 1506)

**DOI:** 10.1186/s12885-017-3451-x

**Published:** 2017-07-03

**Authors:** Qing Zhou, Yong Song, Xin Zhang, Gong-Yan Chen, Dian-Sheng Zhong, Zhuang Yu, Ping Yu, Yi-Ping Zhang, Jian-Hua Chen, Yi Hu, Guo-Sheng Feng, Xia Song, Qiang Shi, Lu Lu Yang, Ping Hai Zhang, Yi-Long Wu

**Affiliations:** 1 Guangdong Lung Cancer Institute, Guangdong General Hospital & Guangdong Academy of Medical Sciences, 106 Zhongshan 2nd Road, Guangzhou, 510080 Guangdong China; 2 Nanjing General Hospital of Nanjing Military Command, Nanjing, Jiangsu China; 3 Zhongshan Hospital, Shanghai, China; 4 The Tumor Hospital affiliated to Harbin Medical University, Harbin, Heilongjiang China; 5 General Hospital of Tianjin Medical University, Heping, Tianjin China; 6 The Affiliated Hospital of Qingdao University, Qingdao, Shandong China; 7 Sichuan Cancer Hospital, Chengdu, Sichuan China; 8 Zhejiang Cancer Hospital, Hangzhou, Zhejiang China; 9 Hunan Cancer Hospital, Changsha, Hunan China; 10 Chinese PLA General Hospital, Beijing, China; 11 The People’s Hospital of Guangxi Zhuang Autonomous Region, Nanning, Guangxi China; 12 Shanxi Cancer Hospital, Taiyuan, Shanxi China; 13 Lilly Suzhou Pharmaceutical Co., Ltd, Shanghai, China

**Keywords:** Chemotherapy, China, Epidermal growth factor receptor, First-line anticancer treatment, Non-small cell lung cancer, Tyrosine kinase inhibitor

## Abstract

**Background:**

In recent years, systemic chemotherapy and molecular targeted therapy have become standard first-line treatments for locally advanced or metastatic nonsquamous non-small cell lung cancer (NSCLC). The objective of this survey was to investigate first-line anticancer treatment patterns and gene aberration test status of patients with advanced nonsquamous NSCLC in China.

**Methods:**

Patients included in this study had unresectable Stage IIIB/IV nonsquamous NSCLC and were admitted during August 2015 to March 2016 into one of 12 tertiary hospitals throughout China for first-line anticancer treatment. Patient data (demographics, NSCLC histologic type, Eastern Cooperative Oncology Group [ECOG] Performance Status [PS], gene aberration test and results [if performed], and first-line anticancer treatment regimen) were extracted from medical charts and entered into Medical Record Abstraction Forms (MERAFs), which were collated for analysis.

**Results:**

Overall, 1041 MERAFs were collected and data from 932 MERAFs were included for analysis. Patients with unresectable Stage IIIB/IV nonsquamous NSCLC had a median age of 59 years, 56.4% were male, 58.2% were never smokers, 95.0% had adenocarcinoma, and 92.9% had an ECOG PS ≤1. A total of 665 (71.4%) patients had gene aberration tests; 46.5% (309/665) had *epidermal growth factor receptor* (*EGFR*) gene mutations, 11.5% (48/416) had *anaplastic lymphoma kinase* (*ALK*) gene fusions, and 0.8% (1/128) had a *c-ros oncogene 1* gene fusion. The most common first-line treatment regimen for unresectable Stage IIIB/IV nonsquamous NSCLC was chemotherapy (72.5%, 676/932), followed by tyrosine kinase inhibitors (TKIs; 26.1%, 243/932), and TKIs plus chemotherapy (1.4%, 13/932). Most chemotherapy regimens were platinum-doublet regimens (93.5%, 631/676) and pemetrexed was the most common nonplatinum chemotherapy-backbone agent (70.2%, 443/631) in platinum-doublet regimens. Most *EGFR* mutation-positive patients (66.3%, 205/309) were treated with EGFR-TKIs.

**Conclusions:**

Findings from our survey of 12 tertiary hospitals throughout China showed an increased rate of gene aberration testing, compared with those rates reported in previous surveys, for patients with advanced nonsquamous NSCLC. In addition, pemetrexed/platinum-doublet chemotherapy was the predominant first-line chemotherapy regimen for this population. Most patients were treated based on their gene aberration test status and results.

**Electronic supplementary material:**

The online version of this article (doi:10.1186/s12885-017-3451-x) contains supplementary material, which is available to authorized users.

## Background

Lung cancer is a major public health concern in China, accounting for 21.3% of all new cancer cases and 27.1% of all deaths caused by cancer in 2012 [1]. Approximately 85% of patients presenting with lung cancer have non-small cell lung cancer (NSCLC) [2], with about 70% of these patients diagnosed with locally advanced or metastatic disease [3]. Recommended first-line treatments for these patients are platinum-doublet chemotherapy or molecular targeted therapy, if sensitive gene aberrations are detected [3–6]. Platinum-doublet chemotherapy has been shown to prolong survival and improve quality of life in patients with advanced NSCLC [4], with comparable efficacy among the various regimens [7]. In NSCLC patients with gene aberrations, molecular targeted therapies have been shown to have greater efficacy and lower toxicity than standard chemotherapy, whereas they have limited efficacy in NSCLC patients without gene aberrations [8].

Findings from a 2010 survey of physicians [9] and a retrospective review of hospital outpatient databases from 2004 to 2013 [10] in China indicated that NSCLC patients were mostly treated with platinum-doublet chemotherapy in the first-line setting. In patients with advanced NSCLC, the most commonly used chemotherapy regimen was gemcitabine/carboplatin-doublet chemotherapy [9, 10]. Of those patients treated with first-line epidermal growth factor receptor (EGFR) tyrosine kinase inhibitors (TKIs), nearly 50% had an unknown or negative *EGFR* mutation status [10]. Reported rates of *EGFR* gene mutation testing in China suggest that only 30% of NSCLC patients with adenocarcinoma are tested for gene aberrations [11] despite more than 40% having *EGFR* mutations [12, 13].

To determine if these practices have changed in recent times, we investigated first-line anticancer treatment patterns and gene aberration test status of patients with unresectable Stage IIIB/IV nonsquamous NSCLC treated at one of 12 tertiary hospitals throughout China.

## Methods

### Study design

This was a survey of medical charts from 12 tertiary hospitals located throughout China (Additional file 1: Table S1). Data were extracted from medical charts of patients discharged from hospital between 1 August 2015 and 15 March 2016.

The protocol was approved by the Research Ethics Committee of the Guangdong General Hospital, Guangzhou, Guangdong, China. Each site obtained its own institutional review board or ethics committee approval before the start of the study. The study was conducted in accordance with the ethical principles of the Declaration of Helsinki and Good Clinical Practice, and was supported by the Chinese Thoracic Oncology Group (CTONG study number 1506).

### Study population

The medical charts of patients meeting the following criteria were included for review: aged ≥18 years; diagnosis of unresectable Stage IIIB or IV (according to the American Joint Committee on Cancer staging system, 7th edition), nonsquamous NSCLC; no previous systemic anticancer treatment for Stage IIIB or IV disease; and most recent hospitalization was for anticancer treatment.

### Study protocol

Data from all patients’ medical charts who met the inclusion criteria were extracted and entered into the Medical Record Abstraction Form (MERAF) by designated hospital staff after patient discharge. Data extracted were demographics, NSCLC histological type, Eastern Cooperative Oncology Group (ECOG) Performance Status (PS), gene aberration test status and results (if performed), and first-line anticancer treatment regimen. Data entry was reviewed on-site by an independent data management organization (Shanghai Centennial Scientific Ltd., Shanghai, China), who assessed accuracy of data entry by checking 20% of all MERAFs collected at one hospital selected at random. Completed MERAFs were collected for analysis.

Data from all collected MERAFs were entered into a database for analysis, with data entered and verified twice to ensure accurate data entry. MERAFs were excluded from analysis if data were missing for gene aberration test status or first-line anticancer treatment regimen and if more than 10% of other data were missing.

### Statistical analysis

Given that there are no published data in China to describe the proportion of patients receiving different types of chemotherapy, we assumed the proportion of patients receiving first-line TKI treatment was stable and could be estimated using the *EGFR* gene mutation rate. The sample size calculation assumed an *EGFR* gene mutation rate of 30% for East Asian populations [11], data from 897 patients to provide 2-sided 95% confidence intervals (CIs) with a precision of 3%, and exclusion of 5 to 10% of MERAFs because of missing data or other errors. Thus, collection of data from 1000 patients was planned.

Data were summarized with descriptive statistics using frequency and percentages for categorical data, and median, minimum, and maximum for continuous data. Analyses were done using SAS® Version 9.3 (SAS Institute, Cary, NC, USA).

## Results

### Patient disposition, demographics, and clinical characteristics

A total of 1041 MERAFs were collected. Of these, 109 MERAFs were excluded because they were of patients who were discharged from hospital before 1 August 2015 (study start date, *n* = 74), had missing data (*n* = 13), or were duplicates (*n* = 22). Thus, 932 MERAFs were included for analysis.

Overall, 73.9% of patients were less than 65 years of age, 56.4% were male, 58.2% had never smoked, 95.0% had adenocarcinoma, and 92.9% had an ECOG PS of 0 or 1 (Table 1).

**Table 1:** Demographics and clinical characteristics of patients with unresectable Stage IIIB/IV nonsquamous non-small cell lung cancer

Characteristic, n (%)	All patients
	*N* = 932
Age, years	
Median (min., max.)	59 (23, 80)
< 65	689 (73.9)
≥ 65	243 (26.1)
Sex	
Male	526 (56.4)
Female	406 (43.6)
Residence area	
Rural	420 (45.1)
Urban	512 (54.9)
Smoking status	
Current Smoker	157 (16.8)
Former Smoker	233 (25.0)
Never Smoker	542 (58.2)
Histologic subtype	
Adenocarcinoma	885 (95.0)
Large Cell Carcinoma	14 (1.5)
Other	33 (3.5)
ECOG PS	
0	291 (31.2)
1	575 (61.7)
2	55 (5.9)
3	11 (1.2)

*ECOG* Eastern Cooperative Oncology Group, *max.* maximum, *min.* minimum, *PS* Performance Status

### Gene aberration test status and results

Overall, 665 (71.4%) patients had gene aberration tests. Gene aberration test rates were 71.4% (665/932) for *EGFR* gene mutations, 44.7% (416/932) for *anaplastic lymphoma kinase* (*ALK*) gene fusions, and 13.7% (128/932) for *c-ros oncogene 1* (*ROS1*) gene fusions. Demographics and clinical characteristics of patients who did and did not have an *EGFR* gene mutation test were similar (Additional file 2: Table S2).

**Fig. 1:**
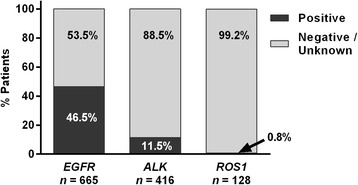
Gene aberration rates of patients with unresectable Stage IIIB/IV nonsquamous non-small cell lung cancer. Patients tested for gene aberrations were classified as positive (activating mutations in exons 18-21), wild type, or unknown (findings inconclusive) for *epidermal growth factor receptor* (*EGFR*) gene mutations, and positive or negative for *anaplastic lymphoma kinase* (*ALK*) and *c-ros oncogene 1* (*ROS1*) gene fusions

Gene aberration rates were 46.5% (309/665) for *EGFR* gene mutations, 11.5% (48/416) for *ALK* gene fusions, 0.8% (1/128) for *ROS1* gene fusions (Fig. 1). Three patients had a co-existing *EGFR* gene mutation and an *ALK* gene fusion. *EGFR* gene mutation rates according to histological subtype were 47.6% (305/641) for adenocarcinoma, 22.2% (2/9) for large cell carcinoma, and 13.3% (2/15) for other histological types. Compared with the overall study population, a numerically higher proportion of *EGFR* mutation-positive patients were female (43.6%, 406/932 vs 56.6%, 175/309, respectively) and had never smoked (58.2%, 542/932 vs 70.2%, 217/309, respectively) (Table 1 and Additional file 2: Table S2).

### First-line anticancer treatment regimens

The predominant first-line anticancer treatment regimen for patients with unresectable Stage IIIB/IV nonsquamous NSCLC was chemotherapy (72.5%, 676/932) followed by TKIs (26.1%, 243/932) and TKIs plus chemotherapy (1.4%, 13/932).

#### Chemotherapy

**Fig. 2:**
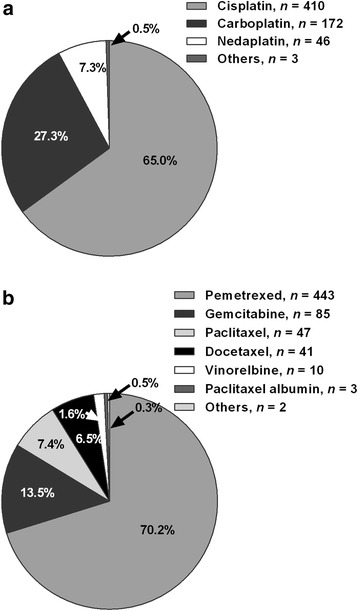
Doublet chemotherapy regimens of patients with unresectable Stage IIIB/IV nonsquamous non-small cell lung cancer, *n* = 631. **a** Platinum agents. **b** Nonplatinum chemotherapy-backbone agents

Most chemotherapy regimens were platinum-doublet regimens (93.3%, 631/676). Platinum-doublet chemotherapy regimens consisted primarily of cisplatin (65.0%) as the platinum agent and pemetrexed (70.2%) as the nonplatinum chemotherapy-backbone agent (Fig. 2).

Other chemotherapy regimens were triplet regimens (platinum-doublet chemotherapy plus bevacizumab; 3.7%, 25/676) and singlet regimens (3.0%, 20/676). Triplet regimens consisted of cisplatin (48.0%, 12/25), carboplatin (32.0%, 8/25), or other platinum agents (20.0%, 5/25) and pemetrexed (60.0%, 15/25), paclitaxel (28.0%, 7/25), gemicitibine (8.0%, 2/25), or docetaxel (4.0%, 1/25) as nonplatinum chemotherapy-backbone agents. Patients treated with triplet regimens were mostly <65 years (80.0%) with an ECOG PS ≤1 (100%) (Additional file 3: Table S3). Singlet regimens consisted of pemetrexed (40.0%, 8/20), docetaxel (30.0%, 6/20), gemcitabine (20.0%, 4/20), or other nonplatinum chemotherapy agents (10.0%, 2/20). Patients treated with singlet regimens were more likely to be ≥65 years (50.0%) and have an ECOG PS ≥2 (30%) (Additional file 3: Table S3).

#### Tyrosine kinase inhibitors

In total, 243 patients with unresectable Stage IIIB/IV nonsquamous NSCLC treated with TKIs. Of these patients, 223 (91.8%) were treated with EGFR-TKIs (gefitinib, erlotinib, icotinib, epitinib, or allitinib), of which 205 (91.9%) were *EGFR* mutation positive. Fewer patients (7.8%, 19/243) were treated with the ALK-TKIs, crizotinib or ceritinib, and 1 (0.4%) patient was treated with the vascular endothelial growth factor receptor (VEGFR)-TKI, apatinib.

#### Tyrosine kinase inhibitors plus chemotherapy

There were 13 patients treated with TKIs plus chemotherapy; 9 patients received EGFR-TKIs plus chemotherapy and 4 patients received ALK-TKIs plus chemotherapy.

### First-line treatment according to gene aberration test status

Most patients with unresectable Stage IIIB/IV nonsquamous NSCLC were treated according to their gene aberration test status (Table 2). A large proportion of patients with *EGFR* gene mutations were treated with EGFR-TKIs (67.0%) and nearly all patients with a negative or unknown gene aberration status were treated with chemotherapy (96.5%). Patients with *ALK* gene fusions were treated with either chemotherapy (56.3%) or ALK-TKIs (35.4%). Three patients with *ALK* gene fusions treated with EGFR-TKIs had co-existing *EGFR* gene mutations.

**Table 2:** First-line anticancer treatment according to gene aberration test status

Treatment, n (%)	Gene aberration				
	*N* = 932				
	Positive			Negative	Unknown
	EGFR^a^	ALK^b^	ROS1	*n* = 307	*n* = 267
	*n* = 309^c^	*n* = 48	*n* = 1		
TKI, *n* = 243	207 (67.0)	20 (41.7)^d^	1	6 (2.0)	9 (3.4)
EGFR,^e^ *n* = 223	205 (66.3)	3 (6.25)^d^	0	6 (2.0)	9 (3.4)
ALK,^f^ *n* = 19	1 (0.3)	17 (35.4)	1	0	0
Chemotherapy,^g^ *n* = 676	95 (30.7)	27 (56.3)	0	296 (96.4)	258 (96.6)
TKI + Chemotherapy, *n* = 13	7 (2.3)	1 (2.1)	0	5 (1.6)	0

*ALK* anaplastic lymphoma kinase, EGFR epidermal growth factor receptor, *ROS1* c-ros oncogene 1, *TKI* tyrosine kinase inhibitor, *VEGFR* vascular endothelial growth factor receptor

^a^ *EGFR* gene mutation positive test included all activating mutations in exons 18-21

^b^ *ALK* tests were determined by fluorescence in situ hybridization, immunohistochemistry, or next-generation sequencing

^c^One patient with an *EGFR* gene mutation was treated with the VEGFR-TKI apatinib

^d^Three patients with *ALK* gene fusions had coexisting *EGFR* gene mutations and were treated with EGFR-TKIs

^e^EGFR-TKIs were gefitinib, erlotinib, icotinib, epitinib, and allitinib

^f^ALK-TKIs were crizotinib and ceritinib

^g^Chemotherapy included singlet, platinum-doublet, and platinum-doublet plus bevacizumab (triplet) regimens

## Discussion

In this survey, we investigated first-line anticancer treatment patterns and gene aberration test status of patients with unresectable Stage IIIB/IV nonsquamous NSCLC at 12 tertiary hospitals throughout China. More than two thirds of patients had gene aberration testing and 46.5% of those tested had *EGFR* gene mutations. The predominant first-line treatment regimen for unresectable Stage IIIB/IV nonsquamous NSCLC was pemetrexed/platinum-doublet chemotherapy. Most patients (66.3%) with *EGFR* gene mutations were treated with first-line EGFR-TKIs. These findings provide an updated and broad overview of the treatment of unresectable Stage IIIB/IV nonsquamous NSCLC in China.

In China, testing for *EGFR* gene mutations is recommended before treating advanced NSCLC [6] and is considered essential for patients with adenocarcinoma given the high rate of *EGFR* gene mutations in East Asian patients [8, 14, 15]. In our survey, 71.4% of patients with unresectable Stage IIIB/IV nonsquamous NSCLC were tested for *EGFR* gene mutations, a rate higher than those reported previously [9, 11]. A 2010 survey of physicians at general hospitals, chest hospitals, and comprehensive cancer centers located in 12 major cities throughout China found only 9.6% of patients with advanced NSCLC (squamous and nonsquamous histology) were tested for *EGFR* gene mutations [9]. Similarly, a 2011 retrospective online survey of patient records found that China had the lowest rate of *EGFR* gene mutation testing of the 6 Asian Pacific countries assessed, with 18.3% of all NSCLC patients and 30.3% of NSCLC patients with adenocarcinoma histology tested [11]. The improved *EGFR* gene mutation test rate in our survey suggests changes in clinical practice since 2010–11, possibly due to increased coverage of testing technology, improved tissue sample collection, and reduced cost. In addition, there may have been less reliance on patient characteristics associated with *EGFR* positive mutations that prompt testing because similar proportions of never smokers versus previous/current smokers (72.5%, 393/542, vs 69.7%, 272/390, respectively) and females versus males (73.9%, 300/406, vs 69.4%, 365/526) had an *EGFR* gene mutation test.

The *EGFR* gene mutation rate detected in our study for all patients (46.5%) and for patients with adenocarcinoma histological subtype (47.6%) were similar to those reported previously for Chinese patients with NSCLC of adenocarcinoma histology (40.3–64.5%) [14]. In a subset analysis of Chinese patients participating in the PIONEER study, a prospective molecular epidemiology study of *EGFR* gene mutations in Asian patients newly diagnosed with advanced NSCLC of adenocarcinoma histology, 50.2% (95% CI: 46.6–53.8%) of patients were *EGFR* mutation positive [12]. In addition, characteristics of *EGFR* mutation-positive patients in our survey were consistent with those associated with higher *EGFR* gene mutation rates (eg, female, never smoker) [16]. The rate of *ALK* gene fusions in our study (11.5%) was slightly higher than those reported previously for Chinese patients with adenocarcinomas (5.1–10%) [14]. Most patients tested for *ALK* gene fusions in our study were *EGFR* wild type, which may have influenced the proportion of patients testing positive for an *ALK* gene fusion because the occurrence of coexisting *EGFR* mutations and ALK gene fusions is rare [17]. The rate of *ROS1* gene fusions (0.8%) was similar to those reported previously (1–2%) in Chinese patients with NSCLC [14].

Platinum-doublet chemotherapy is recommended for treatment of unresectable, advanced NSCLC [6]. Pemetrexed/platinum-doublet chemotherapy was the predominant treatment regimen for unresectable Stage IIIB/IV nonsquamous NSCLC in our survey. In a previous survey of Chinese physicians [9], gemcitabine/platinum-doublet chemotherapy was the predominant regimen for all advanced NSCLC patients (27.4%) and those with adenocarcinoma histology (32.0%). Although a greater proportion of patients with adenocarcinoma were treated with pemetrexed (16.1% vs non-adenocarcinoma 6%, respectively), the prevalence of gemcitabine was attributed to its favorable benefit/toxicity profile, low cost, reimbursement, and low incidence of alopecia [9]. The preference for pemetrexed/platinum-doublet chemotherapy in our survey may be result of changes in physician opinion regarding first-line treatment of unresectable Stage IIIB/IV nonsquamous NSCLC due to increasing evidence of improved overall survival, better tolerability, and fewer toxicities with pemetrexed-doublet regimens than other doublet regimens [18–21] and approval of pemetrexed for first-line treatment of nonsquamous NSCLC in combination with cisplatin by the China Food and Drug Administration in 2014.

Molecular targeted therapy drugs are recommended as first-line treatment options for advanced NSCLC if sensitive *EGFR* gene mutations or *ALK* gene fusions are detected [3–6], because of their higher efficacy and lower toxicity than standard chemotherapy in these patients [8, 22]. In our survey, a large proportion of *EGFR* mutation-positive patients were treated with first-line EGFR-TKIs (66.3%); the remaining *EGFR* mutation-positive patients were treated with chemotherapy (30.7%), EGFR-TKIs plus chemotherapy (2.3%), or other TKIs (0.6%). The reason why more than 30% of *EGFR* mutation-positive patients received chemotherapy only as first-line treatment requires further analysis. Encouragingly, most (91.9%) patients treated with first-line EGFR-TKIs were *EGFR* mutation positive, a proportion higher than that previously reported in a retrospective review of an outpatient oncology database (2004–13) in China (53.5%) [10].

We acknowledge the following limitations of our survey. Our findings from tertiary hospitals may not reflect the situation for those patients being treated at primary or secondary hospitals throughout China. The standard of lung cancer care in China ranges from practices similar to those in Western countries to basic care because China’s large population, expansive geography, and variable socioeconomic status of patients may affect access to diagnostic tests and quality oncology services and treatment [10, 23, 24]. In addition, patients refusing treatment and outpatients were excluded from our survey, which may have introduced bias into our findings. In a retrospective review of an outpatient oncology databases in China [10], 19.1% of patients refused treatment at diagnosis because of poverty, financial insecurity, or lack of medical insurance.

## Conclusion

Our findings from 12 tertiary hospitals located in different geographic areas throughout China provide the most up-to-date overview of treatment patterns and gene aberration test status of patients with unresectable Stage IIIB/IV nonsquamous NSCLC. The rate of gene aberration testing was increased, compared with those rates reported in previous surveys [9, 11], for patients with unresectable Stage IIIB/IV nonsquamous NSCLC. In addition, pemetrexed/platinum-doublet chemotherapy was the predominant first-line regimen for this population and most patients were treated according to their gene aberration test status.

## Additional files

Additional file 1: Table S1. Tertiary hospitals participating in the study. (DOCX 15 kb)

Additional file 2: Table S2. Demographics and clinical characteristics of patients with unresectable Stage IIIB/IV nonsquamous non-small cell lung cancer (NSCLC) according to *epithelial growth factor receptor* (*EGFR*) gene mutation test status and results. (DOCX 18 kb)

Additional file 3: Table S3. Demographics and clinical characteristics of patients with unresectable Stage IIIB/IV nonsquamous non-small cell lung cancer (NSCLC) according to chemotherapy regimen. (DOCX 16 kb)

## Abbreviations

ALK: Anaplastic lymphoma kinase
CI: Confidence interval
CTONG: Chinese Thoracic Oncology Group
ECOG: Eastern Cooperative Oncology Group
EGFR: Epidermal growth factor receptor
max.: Maximum
MERAF: Medical Record Abstraction Form
min.: Minimum
NSCLC: Non-small cell lung cancer
PS: Performance Status
ROS1: c-ros oncogene 1
TKI: Tyrosine kinase inhibitor
VEGFR: Vascular endothelial growth factor receptor
