# Supplementary material for: A multicenter survey of first-line treatment patterns and gene aberration test status of patients with unresectable Stage IIIB/IV nonsquamous non-small cell lung cancer in China (CTONG 1506)
Source: BMC Cancer. 2017 Jul 3;17:462. doi: 10.1186/s12885-017-3451-x (PMC5496179; doi:10.1186/s12885-017-3451-x)
Supplement: Supplementary file 1 — Tertiary hospitals participating in the study. (DOCX 15 kb) [file 12885_2017_3451_MOESM1_ESM.docx]

**Additional file 1: Table S1.** Tertiary hospitals participating in the study

| 1 | 301 Military Hospital | Beijing |
| --- | --- | --- |
| 2 | Guangdong General Hospital | Guangzhou, Guangdong |
| 3 | Hunan Province Tumor Hospital | Changsha, Hunan |
| 4 | Nanjing General Hospital of Nanjing Military Command | Nanjing, Jiangsu |
| 5 | Shanxi Province Cancer Hospital | Taiyuan, Shanxi |
| 6 | Sichuan Cancer Hospital | Chengdu, Sichuan |
| 7 | The Affiliated Hospital of Medical College, Qingdao University | Qingdao, Shandong |
| 8 | The People’s Hospital of Guangxi Zhuang Autonomous Region | Nanning, Guangxi |
| 9 | The Third Clinical School of Harbin Medical University | Harbin, Heilongjiang |
| 10 | Tianjin Medical University Cancer Institute and Hospital | Tianjin |
| 11 | Zhejiang Cancer Hospital | Hangzhou, Zhejiang |
| 12 | Zhongshan Hospital | Shanghai |
