# Supplementary material for: A multicenter survey of first-line treatment patterns and gene aberration test status of patients with unresectable Stage IIIB/IV nonsquamous non-small cell lung cancer in China (CTONG 1506)
Source: BMC Cancer. 2017 Jul 3;17:462. doi: 10.1186/s12885-017-3451-x (PMC5496179; doi:10.1186/s12885-017-3451-x)
Supplement: Supplementary file 2 — Demographics and clinical characteristics of patients with unresectable Stage IIIB/IV nonsquamous non-small cell lung cancer (NSCLC) according to epithelial growth factor receptor (EGFR) gene mutation test status and results. (DOCX 18 kb) [file 12885_2017_3451_MOESM2_ESM.docx]

**Additional file 2: Table S2.** Demographics and clinical characteristics of patients with unresectable Stage IIIB/IV nonsquamous non-small cell lung cancer (NSCLC) according to *epithelial growth factor receptor* (*EGFR*) gene mutation test status and results

|  | ***EGFR* Mutation Test**  ***N* = 932** | | | | |
| --- | --- | --- | --- | --- | --- |
| **Characteristic, n (%)** | **Yes** | | | | **No *n* = 267** |
|  | **Overall**  ***n* = 665** | **Positive^a^  *n* = 309** | **Wild type *n* = 331** | **Unknown**  ***n* = 25** |  |
| Age, years |  |  |  |  |  |
| Median (min., max.) | 59 (23, 80) | 61 (23, 80) | 58 (23, 80) | 56 (37, 68) | 59 (23, 80) |
| <65 | 483 (72.6) | 218 (70.6) | 242 (73.1) | 23 (92.0) | 203 (76.0) |
| ≥65 | 182 (27.4) | 91 (29.5) | 89 (26.9) | 2 (8.0) | 64 (24.0) |
| Sex |  |  |  |  |  |
| Male | 365 (54.9) | 134 (43.4) | 214 (64.7) | 17 (68.0) | 161 (60.3) |
| Female | 300 (45.1) | 175 (56.6) | 117 (35.3) | 8 (32.0) | 106 (39.7) |
| Smoking Status |  |  |  |  |  |
| Current Smoker | 111 (16.7) | 34 (11.0) | 71 (21.4) | 6 (24.0) | 46 (17.2) |
| Former Smoker | 161 (24.2) | 58 (18.8) | 96 (29.0) | 7 (28.0) | 72 (27.0) |
| Never Smoker | 393 (59.1) | 217 (70.2) | 164 (49.6) | 12 (48.0) | 149 (55.8) |
| Histologic Subtype |  |  |  |  |  |
| Adenocarcinoma | 641 (96.4) | 305 (98.7) | 311 (94.0) | 25 (100) | 244 (91.4) |
| Large Cell Carcinoma | 9 (1.4) | 2 (0.7) | 7 (2.1) | 0 (0) | 5 (1.9) |
| Others | 15 (2.3) | 2 (0.7) | 13 (3.9) | 0 (0) | 18 (6.7) |
| ECOG PS |  |  |  |  |  |
| 0, 1 | 620 (93.2) | 289 (93.5) | 308 (93.0) | 23 (92.0) | 246 (92.1) |
| 2, 3 | 45 (6.8) | 20 (6.5) | 23 (7.0) | 2 (8.0) | 21 (7.9) |

ECOG: Eastern Cooperative Oncology Group; max.: maximum; min.: minimum; PS: Performance Status

^a^ *EGFR* gene mutation positive test included all activating mutations in exons 18-21.
