# Supplementary material for: A multicenter survey of first-line treatment patterns and gene aberration test status of patients with unresectable Stage IIIB/IV nonsquamous non-small cell lung cancer in China (CTONG 1506)
Source: BMC Cancer. 2017 Jul 3;17:462. doi: 10.1186/s12885-017-3451-x (PMC5496179; doi:10.1186/s12885-017-3451-x)
Supplement: Supplementary file 3 — Demographics and clinical characteristics of patients with unresectable Stage IIIB/IV nonsquamous non-small cell lung cancer (NSCLC) according to chemotherapy regimen. (DOCX 16 kb) [file 12885_2017_3451_MOESM3_ESM.docx]

**Additional file 3: Table S3**. Demographics and clinical characteristics of patients with unresectable Stage IIIB/IV nonsquamous non-small cell lung cancer (NSCLC) according to chemotherapy regimen

| **Characteristic, n (%)** | **Singlet *n* = 20** | **Doublet *n* = 631** | **Triplet *n* = 25** |
| --- | --- | --- | --- |
| Age, years |  |  |  |
| Median (min., max.) | 65 (35, 79) | 58 (23, 80) | 55 (27, 80) |
| <65 | 10 (50.0) | 478 (75.8) | 20 (80.0) |
| ≥65 | 10 (50.0) | 153 (24.2) | 5 (20.0) |
| Sex |  |  |  |
| Male | 14 (70.0) | 392 (62.1) | 14 (56.0) |
| Female | 6 (30.0) | 239 (37.9) | 11 (44.0) |
| Smoking Status |  |  |  |
| Current Smoker | 5 (25.0) | 123 (19.5) | 3 (12.0) |
| Former Smoker | 5 (25.0) | 174 (27.6) | 4 (16.0) |
| Never Smoker | 10 (50.0) | 334 (52.9) | 18 (72.0) |
| Histologic Subtype |  |  |  |
| Adenocarcinoma | 16 (80.0) | 595 (94.3) | 22 (88.0) |
| Large Cell Carcinoma | 2 (10.0) | 12 (1.9) | 0 (0) |
| Others | 2 (10.0) | 24 (3.8) | 3 (12.0) |
| ECOG PS |  |  |  |
| 0 | 7 (35%) | 200 (31.7) | 7 (28.0) |
| 1 | 7 (35%) | 395 (62.6) | 18 (72.0) |
| 2 | 5 (25%) | 32 (5.1) | 0 (0) |
| 3 | 1 (5 %) | 4 (0.6) | 0 (0) |

ECOG: Eastern Cooperative Oncology Group; max.: maximum; min.: minimum; PS: Performance Status
